# Optimizing tobacco quality and yield through the scientific application of organic-inorganic fertilizer in China: a meta-analysis

**DOI:** 10.3389/fpls.2024.1500544

**Published:** 2024-12-20

**Authors:** Ruixuan Zhu, Shijie He, Huarong Ling, Yongjin Liang, Beilei Wei, Xiaomai Yuan, Wuyang Cheng, Bo Peng, Jinglin Xiao, Jianyu Wei, Yi He, Heyou Xiao, Ziting Wang

**Affiliations:** ^1^ College of Agriculture, University of Guangxi, Nanning, China; ^2^ Guangxi China Tobacco Industry Co., Ltd., Nanning, China; ^3^ Hunan China Tobacco Industry Co., Ltd., Changsha, China; ^4^ Hunan Shaoyang Tobacco Co., Ltd., Shaoyang, China; ^5^ China Key Lab for Conservation and Utilization of Subtropical Agri-Biological Resources, University of Guangxi, Nanning, China

**Keywords:** tobacco, organic-inorganic fertilizer, meta-analysis, yield, quality

## Abstract

China is the largest producer and consumer of tobacco (*Nicotiana tabacum* L.) in the world, and the cultivation and production of tobacco have extremely high economic value and social influence. Applying organic-inorganic fertilizer is a key strategy for boosting tobacco yield and quality. This meta-analysis examines 632 observations from 169 articles to determine the precise influence of organic-inorganic fertilizer on tobacco. It evaluates the effects of different tobacco types and fertilizer compositions on yield and quality after fertilizer application. The application of organic-inorganic fertilizer significantly increased the yield (3.4%), but it mainly improved the balance of chemical composition and enhance the quality of tobacco (high-grade tobacco, 10.3%; reducing sugar content, 5.5%; nicotine content, –5.6%). The Y85 and Y87 varieties showed the most positive response to fertilization, with yield increases of 5.59% and 5.82%, respectively, and high-grade tobacco leaf rates increased by 14.92% and 11.04%, respectively. Fertilizers with a low organic nitrogen ratio (15%-30%) are more effective for increasing yield, while those with a medium to high ratio (50%-60%) improve tobacco’s chemical quality. The application of organic-inorganic fertilizer balanced the nutrient distribution within the tobacco plants, leading to simultaneous increases in both yield and quality. This study quantitatively assessed the effects of organic-inorganic fertilizer on the yield and quality of tobacco and provides a solid theoretical foundation for the scientific and high-quality application of organic-inorganic fertilizer in various tobacco cultivation areas.

## 1 Introduction

Tobacco is one of the most important cash crops in China, and according to the World Health Organization (WHO), China is also the world’s largest producer and consumer of tobacco (https://www.who.int/china/health-topics/tobacco). The cultivation area of tobacco in China had reached 1,000,520 ha by the end of 2022 (National Bureau of Statistics data, https://data.stats.gov.cn/). Meanwhile, India and Brazil, ranking second and third respectively, had cultivation areas of 450,000 ha (India Brand Equity Foundation, https://www.ibef.org) and 261,740 ha (Associação Brasileira dos Produtores de Tabaco, https://afubra.com.br). Therefore, improving the yield and quality of tobacco can create enormous economic benefits in China. Fertilizer is the material basis for increasing tobacco yield, and the rational application of fertilizer is an important measure for improving tobacco yield and quality (Wang et al., 2020). Modern agriculture is characterized by high input, high yield, and high efficiency (Xi et al., 2004), and high crop yields can be achieved with limited arable land and minimal manpower. Continuous and uncontrolled application of fertilizer has become a basic means to increase yield (Guo and Wang, 2021). However, excessive fertilizer use damages land resources, wastes fertilizer, and causes a chemical imbalance in tobacco leaves (Paramesh et al., 2023). The purpose of rational fertilization is not only to increase tobacco yield but also to improve the quality of tobacco leaves.

Organic fertilizer contains a large amount of organic material derived from organic waste, such as animal and plant remain after composting. Compared with inorganic fertilizer, organic fertilizer contains more trace elements and has the ability to regulate the soil structure and improve soil water conservation, fertility, and permeability (Lu et al., 2024; Shang et al., 2020), thereby promoting enzyme and microbial activity in soil. Moreover, the long-term application of organic fertilizer causes less damage to the environment compared with that of inorganic fertilizer (Ouyang et al., 2018). However, the application of organic fertilizers alone leads to a series of problems, such as slower fertilizer release, and in certain regions, higher costs compared to inorganic fertilizer (Panday et al., 2024). It also affects the normal growth and nutrient accumulation of tobacco plants. Therefore, the use of organic-inorganic fertilizer has shown a significant development trend (Yang et al., 2023). Studies have shown that organic-inorganic fertilizer can combine the advantages of organic and inorganic fertilizers thereby improving the yield and quality of tobacco (Bilalis et al., 2010). The correlation between yield increase and quality improvement in tobacco has not been fully established, which may be influenced by numerous factors, including the fertilizer type, chemical composition, and tobacco variety. Gaining a deeper understanding of the relationship between yield increase and quality improvement under the application of organic-inorganic fertilizer is conducive to the continuous and stable increase in the yield, quality, and economic benefits of tobacco crops.

The organic-nitrogen ratio in organic-inorganic fertilizer significantly impacts the fertilization effects on tobacco. A nutrient release rate corresponding to a 25% organic-nitrogen ratio in the fertilizer aligns with the tobacco plant’s growth and development requirements. This alignment is beneficial for enhancing the agronomic indicators, yield, and quality of tobacco, as well as for coordinating the chemical composition within tobacco leaves. However, if the organic nitrogen ratio is too high, the organic-inorganic fertilizer may negatively affect the yield and quality of tobacco. The variety of tobacco is also an important factor that affects the yield of tobacco, and some varieties may show higher yield potential in specific environments because of their genetic characteristics (Tan et al., 2012). Additionally, the variety also affects the chemical composition of tobacco, such as nicotine content, total nitrogen content, reducing sugar, K content, among others (Ma et al., 2018), which directly determine the quality and taste of tobacco (Abubakar et al., 2000; Chen et al., 2017). China has a vast territory, and different planting areas plant different varieties according to their climate and environmental conditions (Sun et al., 2016). Therefore, establishing the quantitative relationship between varieties and changes in tobacco yield and chemical quality after the application of organic-inorganic fertilizer will help tobacco farmers choose the correct organic-inorganic fertilizer to suit their needs.

Several basic field experiments have been carried out in different tobacco planting areas in China to study the different effects of organic-inorganic fertilizer on tobacco. The experiment results of Li et al. (2015) reported that the use of organic-inorganic fertilizer increased the yield (11.4%), output value (18.3%), and high-grade tobacco rate (11%) of the Y97 variety compared with the application of inorganic fertilizer alone, and the authors suggested that the application of organic-inorganic fertilizer with organic-nitrogen ratio of 25-50% was more conducive to the growth and development of tobacco and improved the yield and quality. Ma et al. (2013) found that the application of organic-inorganic fertilizer significantly increased the total sugar content (27.86%), the reducing sugar content (23.00%), sugar-to-nicotine ratio (72.60%), nitrogen-nicotine ratio (22.66%), and K content (6.21%) in tobacco leaves but reduced the total nitrogen content (5.29%) and nicotine (-27.21%), thus leading to a more balanced chemical composition. However, owing to the great differences in climatic conditions, soil physicochemical properties and field management measures in different regions, the experimental results obtained from different studies are inconsistent. To achieve production goals, a suitable organic-inorganic fertilizer ratio scheme should be formulated according to the chemical composition requirements, tobacco varieties, and other factors before planting and fertilization.

Therefore, our study performed a meta-analysis of 169 peer-reviewed studies to (1) identify the specific effects of organic-inorganic fertilizer on tobacco yield and chemical components in tobacco leaves; (2) determine how different tobacco varieties and fertilizer components alter the effects of organic-inorganic fertilizer on tobacco; and (3) reveal the impact of applying organic-inorganic fertilizer on the balance between tobacco yield and quality. This study provides a scientific theoretical reference for improving the fertilization regime of tobacco.

## 2 Materials and methods

### 2.1 Data collection

We searched relevant articles published between 1990-2023 from the China National Knowledge Infrastructure and Web of Science. The search keyword included “flue-cured tobacco” or “tobacco” and “organic fertilizer” or “inorganic fertilizer” and “yield” or “quality” or “chemical composition”. According to the data requirements of the meta-analysis and the purpose of this study, articles were screened using the following criteria: (1) tests in the article should include the application of inorganic fertilizer alone and organic-inorganic fertilizer for comparison; (2) the test materials and environmental background of the test sites should be described (the test sites are located in China); (3) the test results should include the mean and standard deviation of indicators, as these parameters are essential for meta-analysis. (4) the fertilizer treatment section should include the organic-nitrogen ratio; and (5) only one article from the same study can be selected. After screening and evaluation, 169 articles were finally obtained for follow-up analysis.

### 2.2 Data categorization and treatment

**Figure 1:**
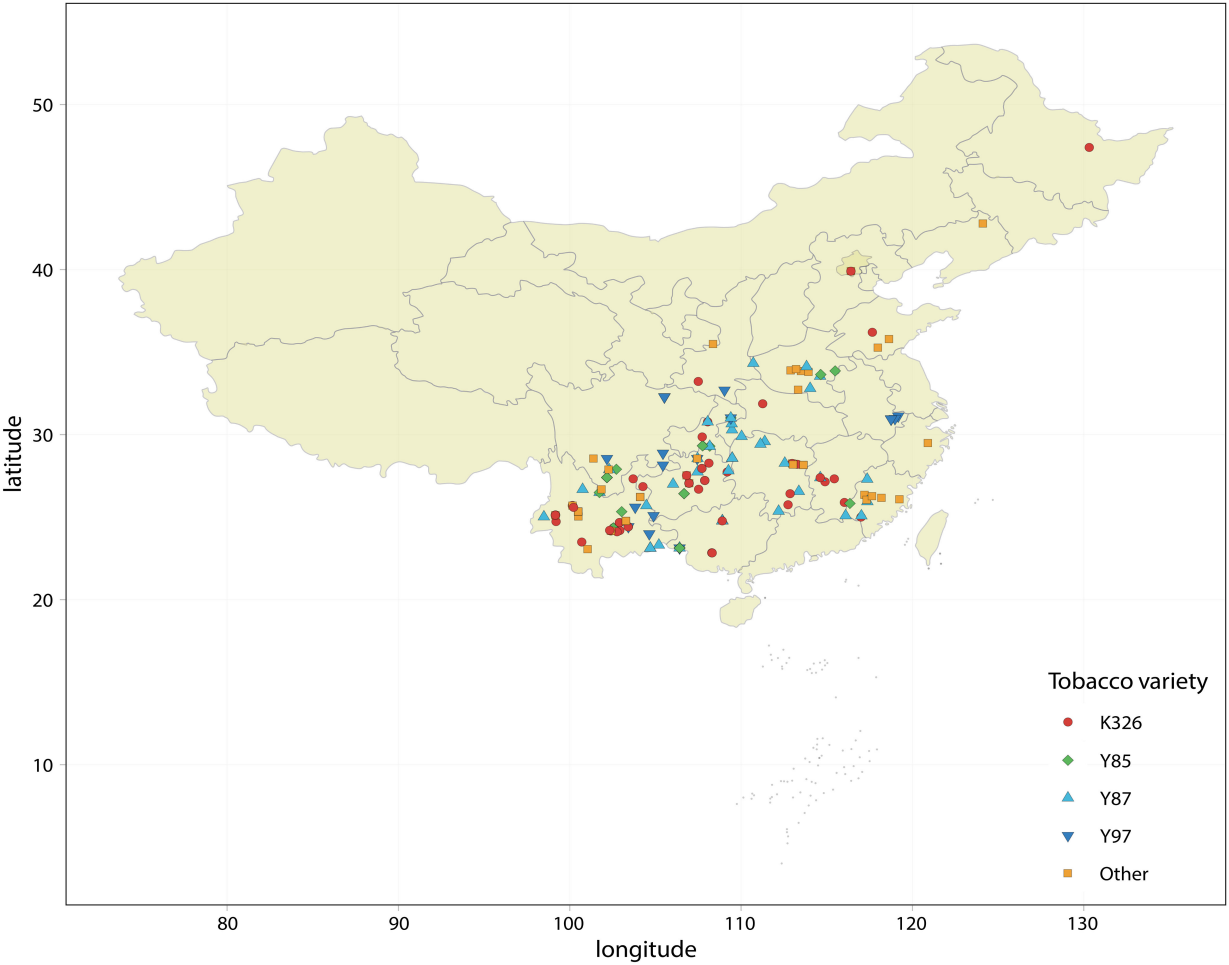
Geographical distribution in China of the tests collected in the meta-analysis.

For each study, we extracted the mean, standard deviation and sample size of tobacco yield, high-grade tobacco rate, output value, total nitrogen content, nicotine content, reducing sugar content, K content and Cl content (the chemical compositions come from the middle leaves of tobacco). These tobacco indicators are the primary subjects of study in Chinese research, reflecting the key aspects of tobacco yield and quality. Extract the mean and standard deviation directly from the article’s tables; use Origin 2023 to extract them from figures; and if only the mean is provided, calculate the standard deviation based on other parameters reported. The following relevant information was collected for analysis: climate conditions (planting site,average annual precipitation, average annual temperature, and average annual sunshine), soil conditions (pH, organic matter content, available nitrogen content, available phosphorus content, and available potassium content), field management measures (planting density, type of organic fertilizer, and organic-nitrogen ratio in mixed fertilizer), and tobacco varieties (K326, Y85, Y87, Y97, and others). A total of 632 sets of observations were selected from drawings and graphs in the 169 articles (**Supplementary Figure S4**). Rosenthal’s fail-safe number was calculated to test publication bias in the studies, if its coefficient >5n + 10 (n is the sample size), then the variable had no publication bias (**Supplementary Table S1**). The location distribution of each experiment in the meta-analysis is shown in **Figure 1**.

### 2.3 Data analysis

Meta-analysis is a quantitative analysis method that summarizes the results of several relatively independent similar studies and draws conclusions (Nakagawa et al., 2023). To better study the effects of organic-inorganic fertilizer on the yield and chemical composition of tobacco and determine the different influences of other factors on the fertilizer’s effects, we performed a meta-analysis of the data in the database and used the log response ratio (lnRR) as the statistical effect value indicator (Gurevitch et al., 2018). Individual lnRRs for each observation were calculated using **Equation 1**:


(1) $$\ln RR=\text{ln}\frac{Y_{e}}{Y_{c}}$$

where Ye and Yc represent the mean values of the treatment and control groups, respectively.

Owing to the large spatiotemporal span of the data in this study and the great differences in planting methods, climate conditions, soil physical-chemical properties in different regions, random effect model (REM) was selected for calculation. The meta-analysis weighted the log response ratio of each observation to obtain the variance (V), weighted factor (Wi), weighted log response ratio (lnRR++), and standard deviation of the weighted log response ratio (SD). They can be calculated using **Equations 2**–**5**:


(2) $$V=\frac{SD_{e}^{2}}{N_{e}Y_{e}^{2}}+\frac{SD_{c}^{2}}{N_{c}Y_{c}^{2}}$$

where $N_{e}\;$ nd $N_{c}$ represent the sample sizes of the treatment and control groups, respectively; and SDe and SDc represent standard deviation for the treatment and control groups, respectively.


(3) $$W_{i}=1/\;\left(V+\tau^{2}\right)$$

(4) $$\text{ln}RR_{++}=\frac{\sum_{i=1}^{k}W_{i}\ln RR}{\sum_{i=1}^{k}W_{i}}$$

(5) $$SD={\left(\frac{1}{\sum_{i=1}^{k}W_{i}}\right)}^{1/2}$$

where i represent the i-th treatment, and k represent the number of observations. $\tau^{2}$ represent the variance between studies due to different studies. Positive values of lnRR*_++_* indicated that the variable increased after the application of organic-inorganic fertilizer, and vice versa.

Equation (6) was used to calculate the 95% confidence interval (CI) of lnRR*_++_*. If the 95% CI did not contain 0, then the application of organic-inorganic fertilizer has a significant impact on this indicators (Du et al., 2020).


(6) $$95\%CI=\text{ln}RR_{++}\;1.96*SD$$

To perceive the rate of change more clearly, lnRR*_++_* and its 95% CI were transformed back to the percentage change, as shown in **Equation 7**.


(7) $$\;E=\text{exp}\left(\text{ln}RR_{++}\right)*100\%-1$$

Data processing and statistical analysis for the meta-analysis were performed using R version 4.3.1 by package “metafor” (Viechtbauer, 2010). Random forest analysis was carried out using the “rfPermute” package in R software, and all images were drawn using the “ggplot2” package in R software. Correlation analysis was performed to examine the pairwise relationships between the lnRR*_++_* of the indicators. Optimal model regression analysis was performed to explain the influence of fertilizer composition on the effect of organic-inorganic fertilizer and the relationship between yield and quality of tobacco. The omnibus test (Qm-test) was used to compare the response of indicators to application of organic-inorganic fertilizer among different subgroups. If the p-value of Qm< 0.05, it suggested a significant effect of this factor on the overall effect (**Supplementary Table S2**).

## 3 Result

### 3.1 Overall effect of organic-inorganic fertilizer on tobacco

**Figure 2:**
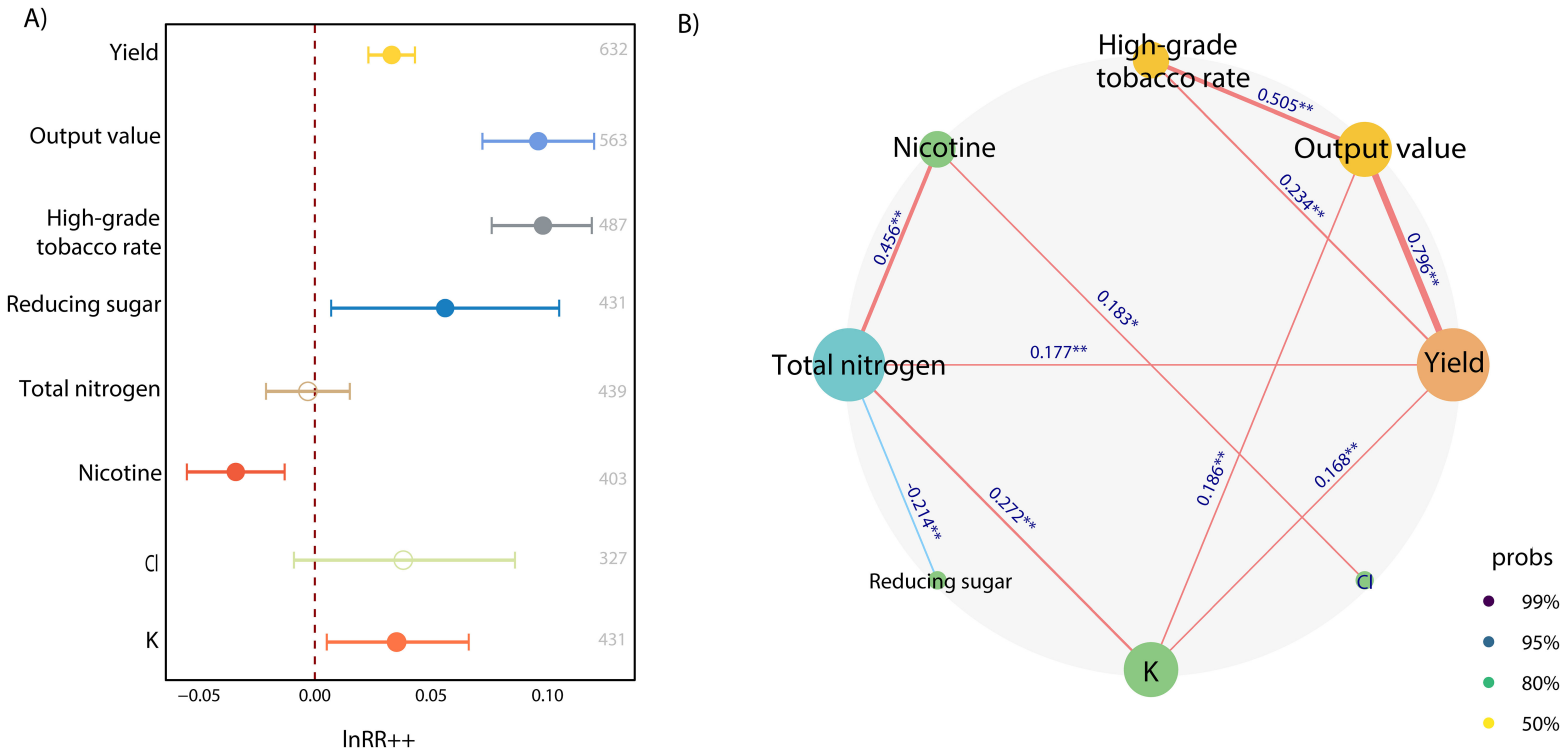
**(A)** Overall effect of organic-inorganic fertilizer on yield and quality of tobacco. The x-axis represents the lnRR*_++_* of the indicators. Error bars represent the 95% confidence intervals. Solid circles represent significant effects and hollow circles indicate insignificant. The Arabic numerals on the right indicate the sample size of an indicator. **(B)** Network correlation analysis of indicator lnRR*_++_*. The numbers on the horizontal axis represent the correlation coefficient. Significant levels at *p< 0.05 and **p< 0.01.

After performing an overall analysis of the 632 sets of data from all 169 studies, we found that the application of organic-inorganic fertilizer significantly increased the yield of tobacco leaves (3.4%), output value (10.1%), high-grade tobacco rate (10.3%), K content (3.76%), and reducing sugar content (5.5%) and significantly decreased the nicotine content (-5.6%) compared with inorganic fertilizer alone (**Figure 2**). However, significant changes were not observed in the Cl or total nitrogen content in tobacco leaves. From the network correlation analysis of indicator lnRR*_++_*, the output value (R=0.796, p<0.01), high-grade tobacco rate (R=0.234, p<0.01), total nitrogen content (R=0.177, p<0.01) and K content (R=0.168, p<0.01) in tobacco leaves was strongly positively correlated with yield. Notably, total nitrogen content and reducing sugar content had significant negative correlations (R=-0.214, p<0.01).

### 3.2 Effect of fertilizer composition on the application of organic-inorganic fertilizer

We collected the organic-nitrogen ratio and the amount of total nitrogen in fertilizers used in different experiments and performed regression analysis of the optimal model with indicators lnRR. As shown in **Supplementary Figure S1** within the range of total nitrogen collected in the study (0-116 kg/hm^2^), the high-grade tobacco rate (p=0.013) and reducing sugar content (p=0.030) in tobacco leaves increased regardless of the total nitrogen, whereas the nicotine content (p=0.087) decreased. The tobacco yield (p<0.001) and output value (p<0.001) only when the amount of total nitrogen exceeded 30 kg/hm^2^. In particular, when the amount of total nitrogen was 50-60 kg/hm^2^, the application of organic-inorganic fertilizer effectively improved the yield, output value and high-grade tobacco rate of tobacco, reduced the nicotine content and increased the content of some chemical components.

**Figure 3:**
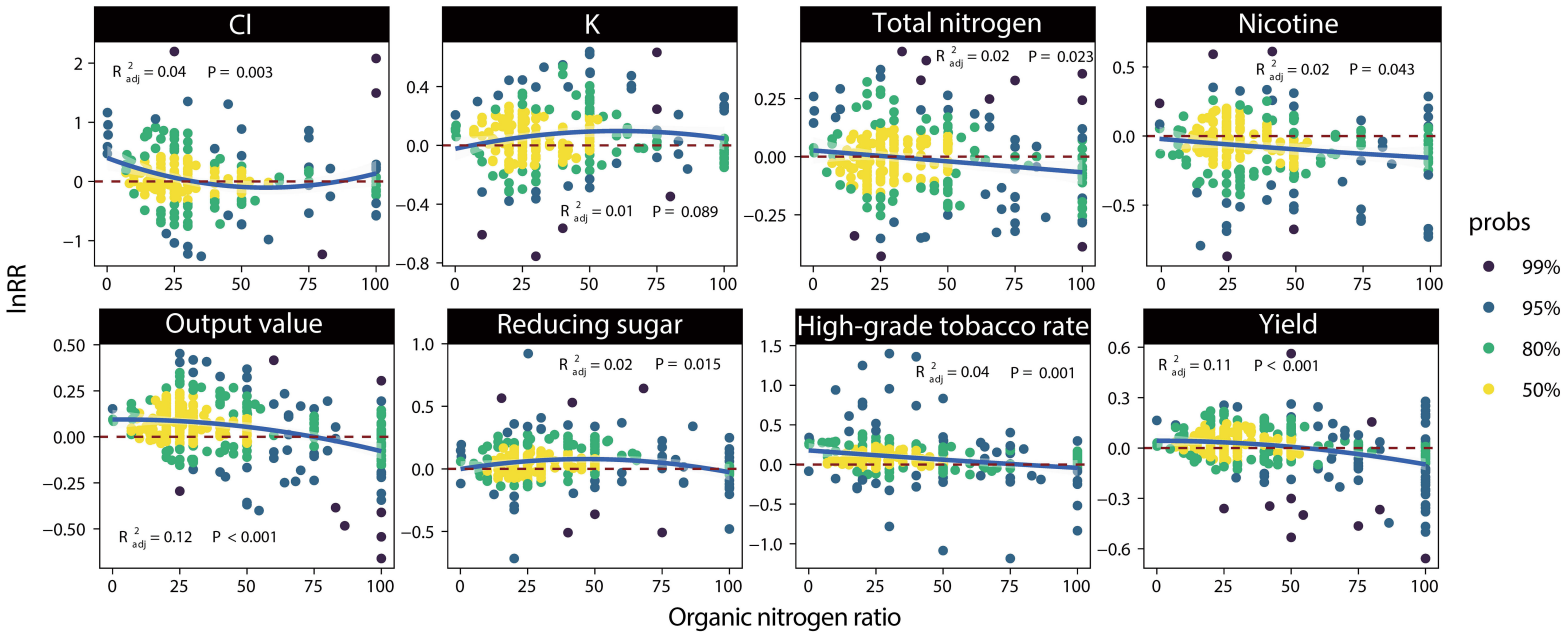
Regression curve of indicators lnRR and organic-nitrogen ratio in fertilizer. Different colored points represent the density of the observed distribution. $R_{adj}^{2}\;$ represent the fitting precision of this regression curve, and p represent the significance of the curve.

For the yield (p<0.001) and output value (p<0.001) of tobacco, within the range of organic-nitrogen ratio collected in the study (7-100%), their lnRR decreased as the organic-nitrogen ratio increased compared with inorganic nitrogen application alone, and the change of Cl content (p=0.003) was similar to yield (**Figure 3**). Notably, the tobacco yield decreased when the organic-nitrogen ratio exceeded 50%. Regarding the chemical indicators in tobacco leaves, we found that nicotine content (p=0.043) and total nitrogen content (p=0.023) decreased as the organic-nitrogen ratio increased. The reducing sugar (p=0.015) and K content (p=0.089) increased regardless of the organic-nitrogen ratio. When the organic-nitrogen ratio in fertilizer was in the range of 50-60%, the reducing sugar and K content showed the greatest increase, and the nicotine content also decreased significantly after fertilization.

### 3.3 Influence significance of subgroups on the application of organic-inorganic fertilizer

We analyzed the influence significance of various factors on the application of organic-inorganic fertilizer, including climatic conditions (planting site, annual average precipitation, annual average temperature, and annual average sunshine), soil conditions (pH, organic matter content, available nitrogen content, available phosphorus content, and available potassium content), planting density and tobacco varieties (**Supplementary Figure S2**). Contrary to our initial expectations, climatic factors had a relatively low impact on the effectiveness of organic-inorganic fertilizer; they were not the primary determinants. In contrast, soil factors showed a more pronounced influence on the application of organic-inorganic fertilizer, with significant differences observed in tobacco yield, high-grade tobacco rate, nicotine content, and total nitrogen content under varying soil conditions. Notably, among the various factors assessed, planting density and tobacco varieties exerted the most significant influence on the application effects of organic-inorganic fertilizer.

### 3.4 Effects of varieties on the application of organic-inorganic fertilizer

**Figure 4:**
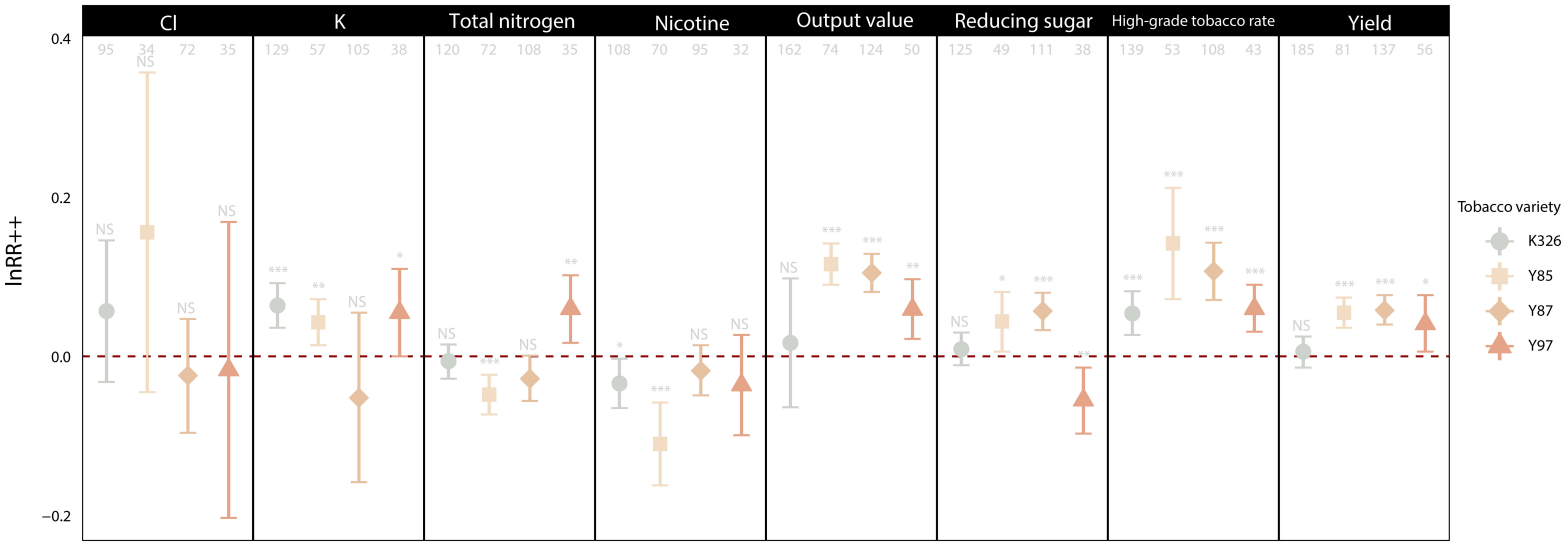
Overall effect of organic-inorganic fertilizer on yield and quality of different varieties tobacco. The y-axis represents the lnRR*_++_* of the indicators. Error bars represent the 95% confidence intervals. Significant levels at NS p>0.05, *p< 0.05, **p< 0.01 and ***p< 0.001.The Arabic numerals on the top indicate the sample size of an indicator.

Four main tobacco varieties (K326, Y85, Y87 and Y97) that have been frequently studied and cultivated in China were selected and analyzed in this study. The yield and quality of K326 tobacco showed a weak in response to organic-inorganic substances, only K content was significantly increased (6.67%, p<0.001). Y85 and Y87 were closely related, their yield (5.59%, p<0.001; 5.82%, p<0.001), high-grade tobacco rate (14.92%, p<0.001; 11.04%, p<0.001), output value (11.94%, p<0.001; 10.78%, p<0.001) and reducing sugar content (4.25%, p<0.05; 5.82%, p<0.001) were significantly increased after applying organic-inorganic fertilizer. For Y97 tobacco, yield (4.03%, p<0.05), high-grade tobacco rate (5.94%, p<0.001), output value (5.71%, p<0.01), K content (5.46%, p<0.05) and total nitrogen content (6.06%, p<0.01) were all significantly increased after applying organic-inorganic fertilizer (**Figure 4**).

**Figure 5:**
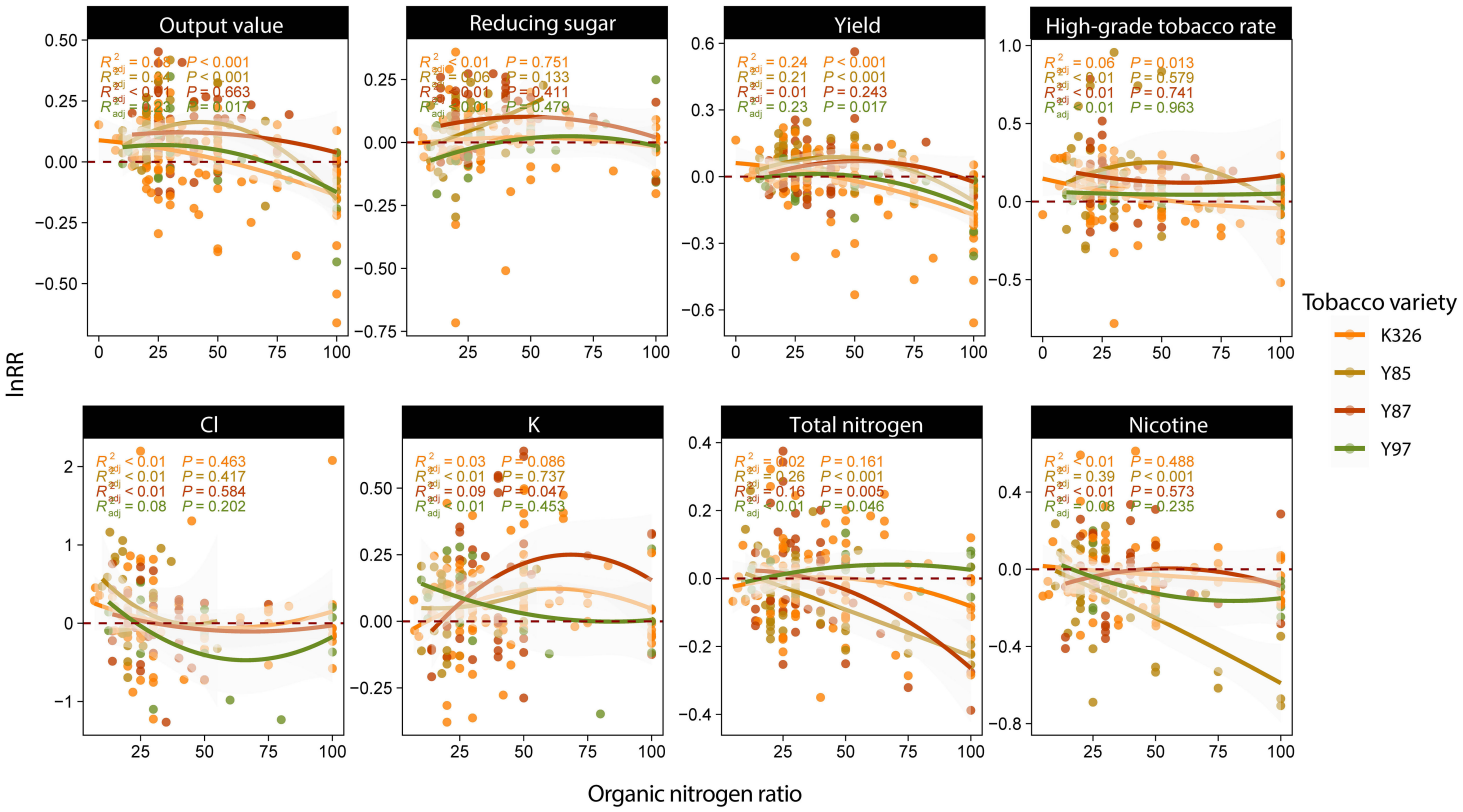
Regression curve of indicators lnRR and organic-nitrogen ratio of different varieties tobacco. The points and curves of different colors represent tobacco varieties. $R_{adj}^{2}\;$ represent the fitting precision of this regression curve, and p represent the significance of the curve.

Regression curves were constructed for the organic nitrogen ratio and indicators lnRR for the four main varieties. For K326, the results showed that organic-inorganic fertilizer with an organic nitrogen ratio below 50% slightly increased the yield (p<0.001), value (p<0.001), and the rate of high-grade tobacco leaves (p=0.013) of K326 tobacco. When the organic nitrogen was about 50%, the K content significantly increased (p=0.083, **Figure 5**). For Y85, yield (p<0.001) and output value (p<0.001) were increased when the organic-nitrogen ratio was less than 80%. Moreover, nicotine content (p<0.001) and total nitrogen content (p<0.001) in Y85 tobacco were significantly affected by the organic-nitrogen ratio. The higher the organic-nitrogen ratio, the greater the reduction in nicotine and total nitrogen (**Figure 5**).

### 3.5 Relationship between yield and quality of different tobacco varieties after applying organic-inorganic fertilizer

**Figure 6:**
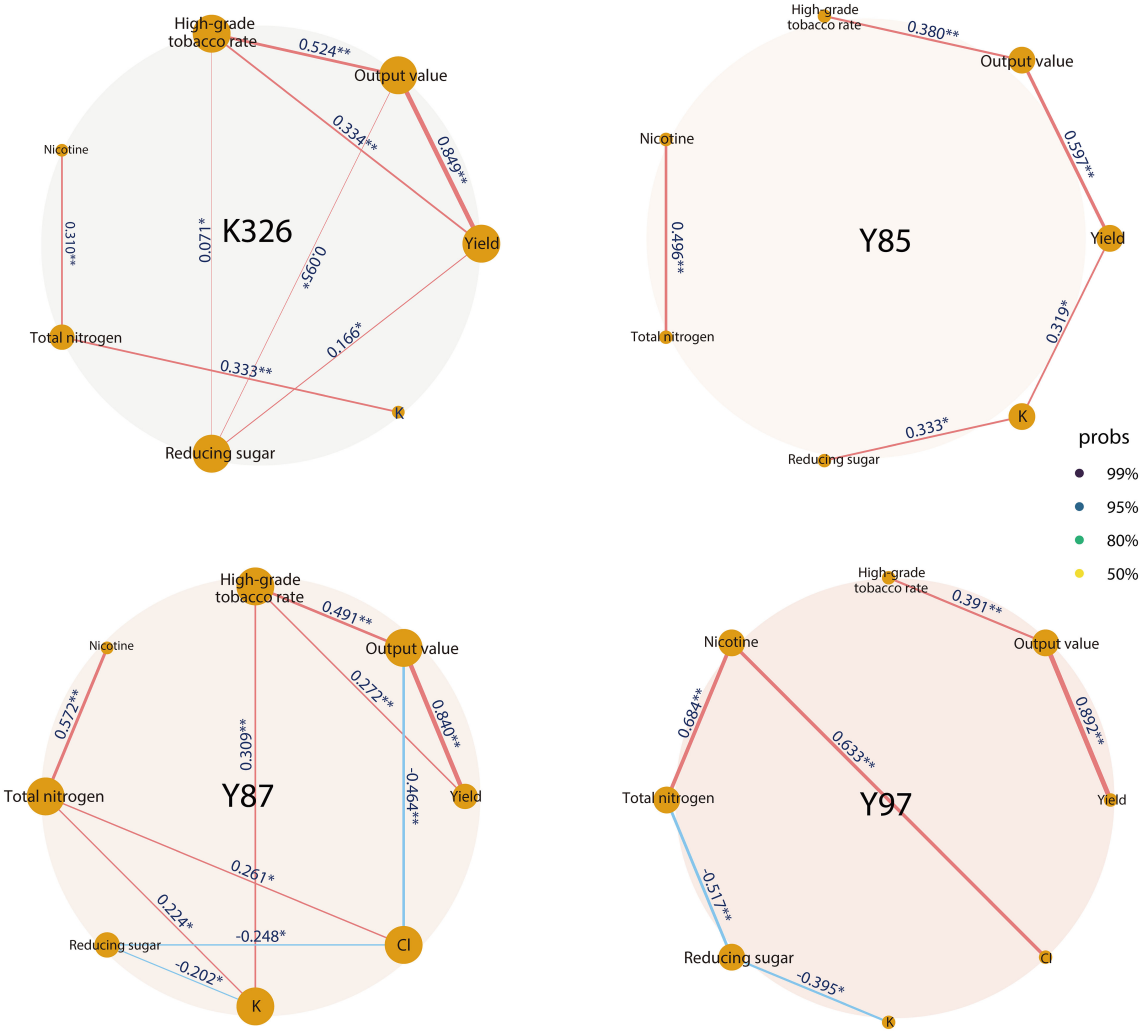
Network correlation analysis of lnRR*_++_* of indicators of different tobacco varieties. The numbers on the horizontal axis represent the correlation coefficient. Significant levels at *p< 0.05, **p< 0.01 and ***p< 0.001.

As shown in **Figure 6**, among the four varieties, K326 and Y87 showed strong correlations with each indicator while Y85 showed the weakest correlations. The output value of all varieties was positively correlated with the yield (p<0.01), high-grade tobacco rate (p<0.01), and the total nitrogen content was positively correlated with the nicotine content (p<0.01). Reducing sugar content was positively correlated with yield (R=0.166, p<0.05), output value (R=0.095, p<0.05), and high-grade tobacco rate (R=0.071, p<0.05) in K326 tobacco.

The yield of Y85 was positively correlated with the K content (R=0.319, p<0.05), and the K content was positively correlated with the reducing sugar content (R=0.333, p<0.05). In Y87, increase in the K content significantly increased the high-grade tobacco rate (R=0.309, p<0.01).

**Figure 7:**
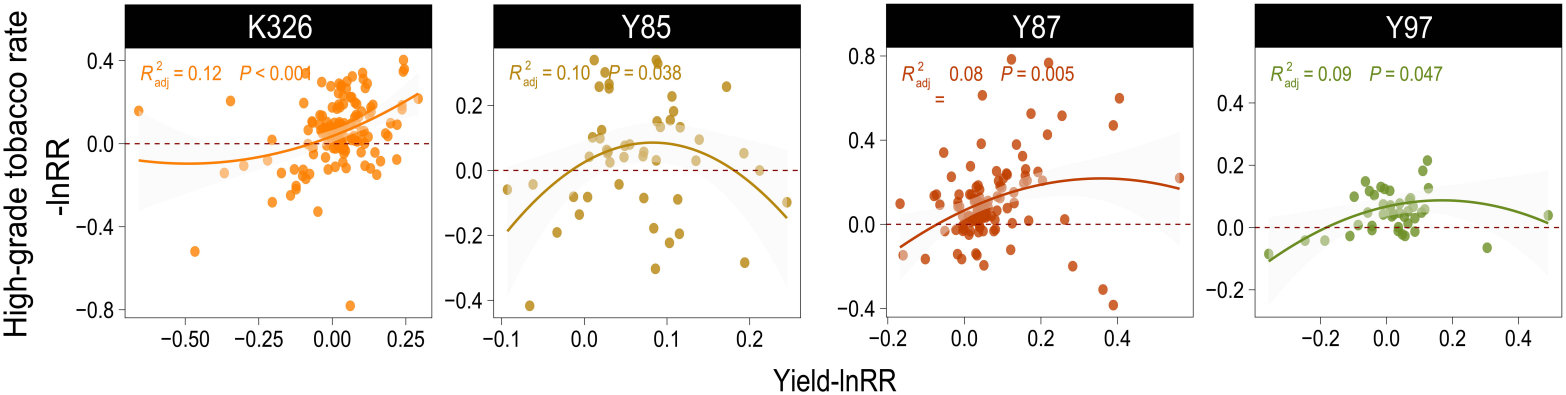
Regression curve between yield of lnRR different varieties and high-grade tobacco rate lnRR. The points and curves of different colors represent tobacco varieties. $R_{adj}^{2}\;$ represent the fitting precision of this regression curve, and p represent the significance of the curve.

**Figure 7**; **Supplementary Figure S3** show the regression relationship between yield and other indicators of four varieties after applying organic-inorganic fertilizer. With an increase of yield, the high-grade tobacco rate (p<0.001, p=0.038, p=0.005, p=0.047) and output value (p<0.001, p<0.001, p<0.001, p<0.001) of the four varieties increased. Organic-inorganic fertilizer application can simultaneously improve yield and quality. However, except for K326, the curve between the high-grade tobacco rate and yield of the other three varieties is similar to a parabola, which means that the increase in the high-grade tobacco rate become declined when the yield increased to a certain extent and even had a negative effect. In Y85, K content (p=0.010) and reducing sugar content (p=0.045) increased while the total nitrogen (p=0.045) and nicotine (p<0.001) content significantly decreased as the yield increased (**Supplementary Figure S3**).

## 4 Discussion

### 4.1 Effects of application of organic-inorganic fertilizer on tobacco yield and quality

We found that the yield, output value and high-grade tobacco rate of tobacco were significantly increased after applying organic-inorganic fertilizer, and the increase in yield also affected the increase in output value and high-grade tobacco rate (**Figure 2A**). Tobacco has strict nitrogen requirements at different growth stages (Rosa et al., 2006). In the early growth stage, sufficient nitrogen is required to maintain the full growth of tobacco until flowering, and a high nitrogen supply is not subsequently required. The application of inorganic fertilizer alone provided nutrients required by tobacco in the early stage. Compared with inorganic fertilizer, the release of nutrients in organic fertilizer is slower (Peng et al., 2014). Therefore, the application of organic-inorganic fertilizer can provide tobacco with an appropriate nutrient supply during the entire growth and development period, thereby ensuring the nutrient absorption and growth of tobacco. Moreover, organic fertilizer can improve the plant root environment, enrich the types of soil microorganisms, enhance the activity of soil extracellular enzymes (Jiang et al., 2022), and improve the content of nutrient element in the soil, which contributes to an increase in the tobacco yield and high-grade tobacco rate. However, compared with the increase of output value and high-grade tobacco rate, the increase of yield was small, indicating that the main effect of the application of organic-inorganic fertilizer on tobacco was not to increase yield but to improve the quality of tobacco (**Figure 2A**).

Superior quality tobacco exhibits a balance and coordination between carbohydrates and nitrogen compounds (Banožić et al., 2020). Compared with inorganic fertilizer alone, the application of organic-inorganic fertilizer significantly increased the reducing sugar content in tobacco (**Figure 2A**). Organic -inorganic fertilizer can improve soil invertase activity (Song et al., 2022), and the invertase in the rhizosphere soil of tobacco can split disaccharides, increase the available carbon content in the soil, and promote the tobacco to absorb and use carbon to synthesize carbohydrates. Meanwhile, the application of organic fertilizer brought a large amount of humic acid to the soil (Li et al., 2008). Humic acid can affect the physiological metabolism of tobacco by promoting root growth and nutrient absorption, thereby increasing the accumulation of reducing sugar content (Ampong et al., 2022). Organic-inorganic fertilizer also reduced nicotine content in tobacco (**Figure 2A**), and increased the sugar-to-nicotine ratio, which shifted the sugar-to-nicotine ratio to the high-quality range. The application of organic-inorganic fertilizer improved the situation of excessive N supply from inorganic fertilizer and slow N supply from organic fertilizer, making the N supply from fertilizer more stable and lasting. Nicotine and total nitrogen were positively correlated in the tobacco plants (**Figure 2B**), thereby, adjusting the nitrogen was also conducive to the decrease of nicotine content (Liu et al., 2020; Fu et al., 2016). In general, organic-inorganic fertilizer has been found to harmonize the carbon-nitrogen relationship in tobacco and enhance tobacco quality.

### 4.2 Effect of fertilizer composition on yield and quality of tobacco

As mentioned above, the absorption of organic and inorganic fertilizers by tobacco is related to different growth periods. Therefore, if the organic-nitrogen ratio in organic-inorganic fertilizer is unbalanced, the content of available nitrogen in the soil during the early stages will be lower and the yield will be reduced (Suchoff et al., 2020; Xi et al., 2005). we found that organic-inorganic fertilizer with low organic-nitrogen ratio could increase tobacco yield, and when the organic-nitrogen ratio exceeded 50%, organic-inorganic fertilizer would reduce tobacco yield (**Figure 3**). The organic-nitrogen ratio can affect tobacco yield by controlling the synthesis and degradation of chlorophyll in leaves. Organic-inorganic fertilizer with 15%-30% organic nitrogen increases the chlorophyll content of tobacco leaves in the early growth stage, ensuring the normal degradation of chlorophyll in the later stage, and show normal yellowing maturation, which enhances the photosynthetic rate and promote the accumulation of tobacco dry matter, thereby helping to increase the yield of tobacco leaves. Whereas when organic nitrogen is more than 45%, the chlorophyll cannot be degraded normally at maturity, thus causing late ripening of tobacco and reducing the yield (Zhou et al., 2009). The yield and output value required the amount of total nitrogen to exceed 30 kg/hm^2^ to increase in **Supplementary Figure S1**, which also confirms our thesis.

When the ratio of organic nitrogen was within the range of 50-60% and the amount of total nitrogen was controlled within 50-60 kg/hm^2^, organic-inorganic fertilizer had the best effect on coordinating the chemical composition (**Supplementary Figures S1**, **3**). At this ratio, organic-inorganic fertilizer can enhance the C metabolism in the sugar accumulation period and effectively regulate the N metabolism of tobacco (Tang et al., 2010), which is conducive to the coordinated development of carbon and nitrogen and the improvement of quality of tobacco. Liu et al. (2018) also found that when the organic nitrogen substitution ratio was 50%, the abundance of beneficial bacteria in soil could be maximized. These beneficial bacteria not only participate in the decomposition of soil organic matter and also accelerate the soil nitrogen cycle (Meng et al., 2022), but also directly carry out biological nitrogen fixation, carbon fixation, oxygen increase and perform other physiological activities to improve soil fertility (Álvarez et al., 2023). Beneficial bacteria help tobacco roots to better absorb and utilize nutrients and coordinate the chemical composition of tobacco leaves. In summary, we believe that the ratio of organic nitrogen applicable to increasing yield and coordinating chemical composition of tobacco is different. Only a small ratio of organic nitrogen can achieve a good yield-increasing effect, whereas a higher ratio of organic nitrogen is required to improve the chemical composition. This finding may play a guiding role in the scientific fertilization of tobacco and improving its economic benefits.

### 4.3 Effects of application of organic-inorganic fertilizer on yield and quality of different tobacco varieties

Different tobacco varieties responded differently to the application of organic-inorganic fertilizer. The effect was the best in Y85 and Y87 varieties, followed by Y97, and was least effective in K326 (**Figures 4**, **5**, **7**). This variation is mainly attributed to differences in growth habits, genetic characteristics, root distribution, and nutrient requirements. K326 is widely planted in various regions of China (**Figure 1**). Observations of the microstructure of the tobacco leaf tissues and stomata showed that K326 had a balanced leaf thickness, tissue, and stomatal structure and good ecological adaptability, making it suitable for planting in most environments. Our study suggests that K326 can absorb sufficient nutrients for normal growth and development even with less effective fertilizers because of its good adaptation and resilience (Zhou, 2005; Pandey et al., 2021). Therefore, the yield and most chemical components of this variety did not change significantly after the application of organic-inorganic fertilizer, while only the K content in tobacco leaves increased significantly. Organic-inorganic fertilizer can promote the synthesis of more K in tobacco. The K content of K326 was approximately 2%, higher than that of the other varieties. More nutrients related to K synthesis need to be absorbed during growth and development (Luo et al., 2014). Consequently, the K content of K326 increased significantly after the application of organic-inorganic fertilizer, with an organic-nitrogen ratio of approximately 50% being particularly beneficial for enhancing K content in K326 (**Figure 7**). The yield and output value of Y85 and Y87 responded positively to organic-inorganic fertilizer (**Figure 4**). Chen et al., 2024 found that the leaf growth and dry matter accumulation rate of Y87 and Y85 were slower than those of K326 and other varieties. Thus, the application of organic-inorganic fertilizer is particularly beneficial for accelerating yield formation in these varieties. Compared with Y87, total nitrogen content and nicotine content in Y85 were more affected by organic-inorganic fertilizer (**Figure 4**). Total nitrogen and nicotine contents decreased significantly and continued to decline with an increasing proportion of organic nitrogen. Nicotine content in roasted tobacco leaves can neutralize the acidic substances produced by the burning of carbohydrates, which is conducive to the formation of a good taste (Sun et al., 2012), but when its content exceeds a certain range, it will have a certain negative impact on the taste (Wu and Wang, 2010). The nicotine content in Y85 tobacco leaves was higher compared to other varieties (Wu et al., 2010). The application of organic-inorganic fertilizer can reduce the nicotine content to a certain extent, which is helpful to improve the sensory quality of this variety. In conclusion, the application of organic-inorganic fertilizer significantly increased the K content of K326, improved the yield and quality of Y87 and Y85, effectively reduced the total nitrogen content and nicotine content of Y85 tobacco leaves, and coordinated the chemical composition of Y85 better.

### 4.4 Relationship between yield and quality of tobacco after applying organic-inorganic fertilizer

Due to the limited nutrients within crop, increasing crop yield and improving crop quality are two aspects that are closely related but contradictory. The high-grade tobacco rate is the ratio of tobacco leaves rated as superior relative to the total number of tobacco leaves. This parameter comprehensively considers the agronomic indicators, chemical quality, and sensory quality of tobacco, and its level directly reflects the quality of the tobacco. We found that after the application of organic-inorganic fertilizer, the yield and high-grade tobacco rate of the four tobacco varieties increased to different degrees (**Figure 4**). However, the high-grade tobacco rate does not continue to increase with an increase in yield, and when the yield increase reaches a certain value, the increase in high-grade tobacco rate begins to decline. When the yield is too high, plants use more energy and nutrients for the growth of stems and leaves, thus ignoring the formation of tobacco quality substances (Raza et al., 2017). Wang et al. (2009) also found that the relationship between tobacco yield and quality was similar to a parabola. When the yield ranged from 2040 kg/hm^2^ to 2 775 kg/hm^2^, the quality of tobacco increased; however, after the yield exceeded 2775 kg/hm^2^, the quality of tobacco leaves began to deteriorate.

The K and reducing sugar content in Y85 were positively correlated with the yield (**Figure 6**), and they both increased with an increase in yield (**Supplementary Figure S3**). This is consistent with our expectations, because the essence of the increase in tobacco yield is that the leaves become larger and thicker, and the leaf area increases, which is more conducive to the photosynthesis. Photosynthesis is the main method of accumulating carbohydrates in plants, and an increase in photosynthesis increase the reducing sugar content (Muhie, 2022). K participates in the movement of leaves and stomata and is an important cation that promotes the synthesis and transportation of photosynthetic and assimilation products. K deficiency will enhance stomatal and mesophyll resistance, and reduce the absorption of CO^2^ at the leaf surface (Hafsi et al., 2014; Ho et al., 2020; Lu et al., 2019). Therefore, an increase in yield must be accompanied by an increase in photosynthesis, K content and reducing sugar content in Y85 tobacco leaves. Bilalis et al. (2015) found that plants need more nutrients to be transported to the leaves when the tobacco yield increases, which leads to the redistribution of some nutrients and the removal of some alkaloid substances from the roots. Total nitrogen and nicotine in the tobacco leaves may be reduced or transferred to other parts of the tobacco plant. This is consistent with the results of the present study, in where we found that an increase in yield led to a significant decrease in total nitrogen in Y85 tobacco leaves and a decrease in nicotine content (**Supplementary Figure S3**). In conclusion, organic-inorganic fertilizer has the potential to coordinate the distribution of nutrients within tobacco plants and simultaneously improve yield and quality. However, further research is needed to achieve this goal.

## 5 Conclusions

Our meta-analysis results showed that although the application of organic-inorganic fertilizer improved the yield of tobacco, the main effect was to improve the balance of the chemical composition and improve the quality of tobacco. Second, by analyzing the effects of organic-inorganic fertilizer components on the application effect, we concluded that organic-inorganic fertilizer with a low ratio of organic nitrogen (15–30%) was more beneficial for increasing tobacco yield while fertilizer with a medium and high ratio of organic nitrogen (50–60%) had a better effect on improving tobacco chemical quality. Application of organic-inorganic fertilizer had the best effect on Y85 and Y87 and improved the yield and quality, and it also effectively reduced the total nitrogen and nicotine content of Y85 tobacco leaves. It had the worst effect on K326, which only showed an increase in the K content. This study also concluded that organic-inorganic fertilizer simultaneously increased the yield and high-grade tobacco rate of the four main varieties under certain conditions. Moreover, organic-inorganic fertilizer also increased the reducing sugar and K content, reduced the nicotine content in Y85 while increasing the yield.

## Data Availability

The raw data supporting the conclusions of this article will be made available by the authors, without undue reservation.
