# Supplementary material for: Optimizing tobacco quality and yield through the scientific application of organic-inorganic fertilizer in China: a meta-analysis
Source: Front Plant Sci. 2024 Dec 20;15:1500544. doi: 10.3389/fpls.2024.1500544 (PMC11697594; doi:10.3389/fpls.2024.1500544)
Supplement: Supplementary file 2 [file DataSheet1.docx]

Fig. S1. Regression curve of indicators lnRR and total inorganic nitrogen in fertilizer. Different colored points represent the density of the observed distribution. $R_{adj}^{2}$represent the fitting precision of this regression curve, and p represent the significance of the curve.





Fig. S2. Prediction of the importance of different factors on the application of organic-inorganic fertilizer by forest analysis. Colors represent the importance of different factors to the application of organic-inorganic fertilizer. PS=planting site; AAP= annual average precipitation; AAT= annual average temperature; AAS= annual average sunshine; PD=planting density; TV=tobacco variety; AP= available phosphorus; AN= available nitrogen; OMC= organic matter content; AK= available potassium.





Fig. S3. Regression curve between yield lnRR and other indicators lnRR of different varieties. $R_{adj}^{2}$represent the fitting precision of this regression curve, and p represent the significance of the curve.


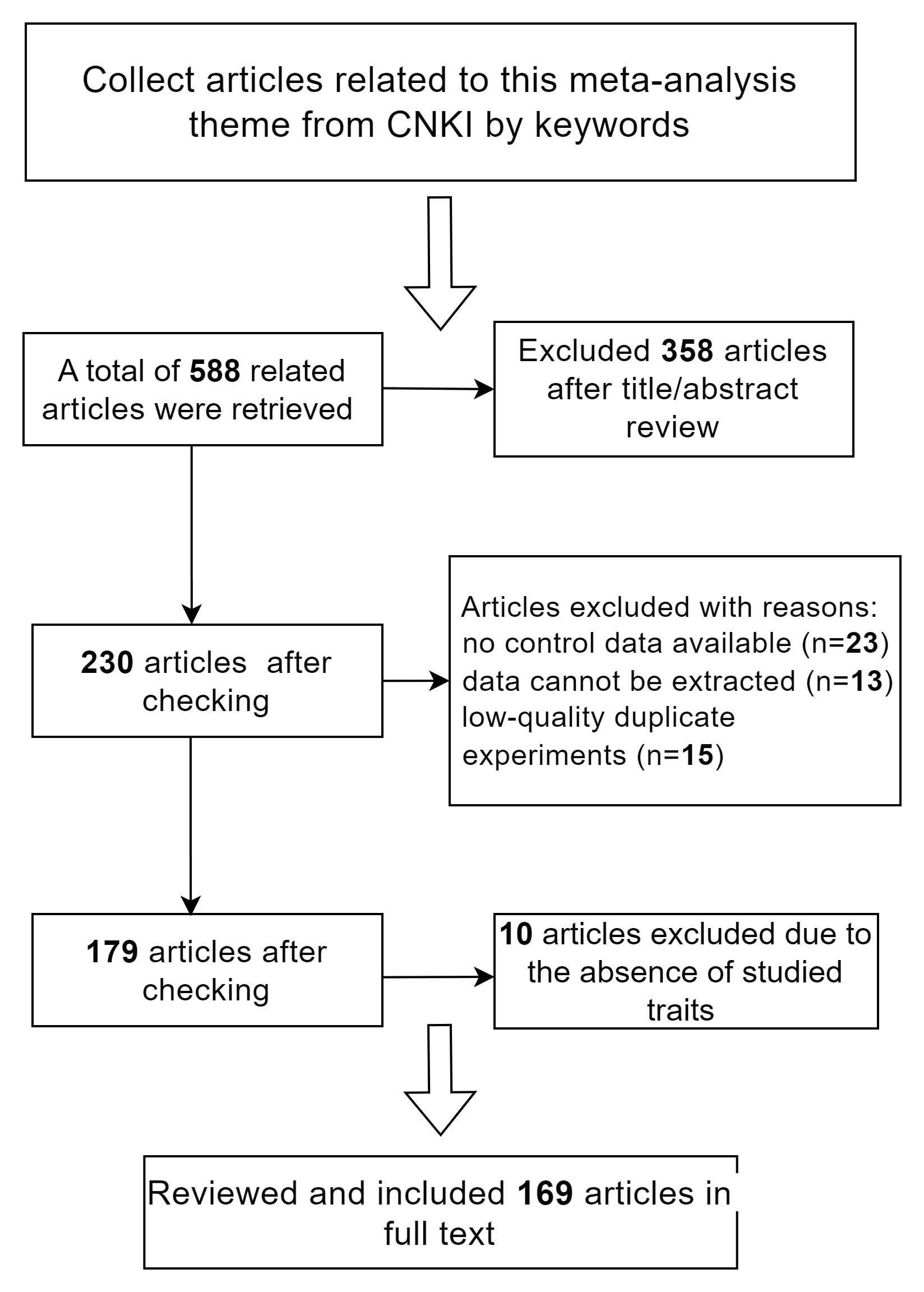


Fig. S4. PRISMA flow diagram showing the procedure used for selection of studies for synthesis.

Table.S1. Results of the analyses for publication bias. Rosenthal’s fail-safe number of an indicator is larger than 5n+10 (n is the sample size) suggest there was no publication bias.

| Indicators | 5n+10 | Fail safe number | p-val |
| --- | --- | --- | --- |
| Yield | 3170 | 96004 | <0.001 |
| Output value | 2825 | 237930 | <0.001 |
| High-grade tobacco rate | 2445 | 500190 | <0.001 |
| Nicotine | 2025 | 119518 | <0.001 |
| Total nitrogen | 2205 | 64570 | <0.01 |
| Reducing sugar | 2165 | 70962 | <0.001 |
| K | 2165 | 33912 | <0.001 |
| Cl | 1645 | 15257 | <0.001 |

Table. S2. Qm-test values and significance of different subgroups. Colors ranging from dark to light represent the significance levels of P-values, with the darkest indicating P < 0.001, followed by P < 0.01, P < 0.05, and the lightest representing non-significant results. PS=planting site; AAP= annual average precipitation; AAT= annual average temperature; AAS= annual average sunshine; ONR=Organic nitrogen ratio; PD=planting density; TV=tobacco variety; AP= available phosphorus; AN= available nitrogen; OMC= organic matter content; AK= available potassium.

| Qm-test values | Yield | Output value | High-grade tobacco rate | Nicotine | Total nitrogen | Reducing sugar | K | Cl |
| --- | --- | --- | --- | --- | --- | --- | --- | --- |
| PS | 2.412 | 0.990 | 0.026 | 2.603 | 0.368 | 0.381 | 2.240 | 1.929 |
| AAP | 1.895 | 0.931 | 1.768 | 1.281 | 0.921 | 1.135 | 0.983 | 2.468 |
| AAT | 2.727 | 2.849 | 2.180 | 1.795 | 1.154 | 1.296 | 0.836 | 3.283 |
| AAS | 2.019 | 0.595 | 1.458 | 1.667 | 1.013 | 0.483 | 5.355 | 2.444 |
| ONR | 8.822 | 8.796 | 4.956 | 7.650 | 2.966 | 9.489 | 7.920 | 39.539 |
| PD | 1.977 | 51.544 | 17.941 | 5.100 | 1.014 | 7.570 | 3.673 | 30.493 |
| TV | 4.586 | 4.657 | 62.804 | 4.545 | 1.316 | 1.907 | 1.522 | 25.129 |
| pH | 3.701 | 1.775 | 0.765 | 3.163 | 1.676 | 6.513 | 3.697 | 2.339 |
| AP | 2.632 | 5.167 | 0.635 | 1.730 | 0.801 | 5.746 | 4.295 | 0.980 |
| AN | 2.762 | 5.283 | 0.632 | 2.347 | 1.480 | 4.409 | 3.883 | 2.108 |
| OMC | 2.880 | 5.595 | 0.699 | 2.741 | 1.428 | 5.652 | 0.978 | 2.072 |
| AK | 2.852 | 3.222 | 0.636 | 1.846 | 1.220 | 5.845 | 4.065 | 1.062 |
